# EEG microstate responses of migraine patients to transcutaneous electrical nerve stimulation

**DOI:** 10.3389/fneur.2026.1883163

**Published:** 2026-07-29

**Authors:** Qi Liu, Yang Guo, Shuyong Jia, Xiaoyu Wang, Yonglie Zhao, Jingjing Wang, Yulong Ding, Jie Ji, Na Tu, Qiuyue Lyu, Yuhan Liu, Zixin Huo, Xiaojing Song, Shuyou Wang, Weibo Zhang, Yi Guo, Guangjun Wang

**Affiliations:** 1 Institute of Acupuncture and Moxibustion, China Academy of Chinese Medical Sciences, Beijing, China; 2 Tianjin University of Traditional Chinese Medicine, Tianjin, China; 3 National Clinical Research Center for Chinese Medicine, Tianjin, China; 4 First Teaching Hospital of Tianjin University of Traditional Chinese Medicine, Tianjin, China; 5 Beijing University of Chinese Medicine Third Affiliated Hospital, Beijing, China; 6 Beijing Fengtai Hospital of Integrated Traditional and Western Medicine, Beijing, China; 7 Tsinghua University Yuquan Hospital, Beijing, China

**Keywords:** acupuncture, EEG, microstate, migraine, transcutaneous electrical nerve stimulation (TENS)

## Abstract

**Background:**

Previous studies have revealed that migraine patients have unique alterations in their electroencephalography (EEG) microstates; however, no studies have explored the neural responses of migraine patients to therapeutic treatment through EEG microstate analysis. This study applied EEG microstate analysis and transcutaneous electrical nerve stimulation (TENS) to explore the neural responses of migraine patients to therapeutic treatment.

**Methods:**

Fifty-one migraine patients with or without aura were recruited and randomly divided into TENS (patients were stimulated with half of the maximum tolerable current) or sham TENS (patients were stimulated with 1 mA) groups. Patients underwent resting-state EEG examinations. The EEG microstate parameters of four widely recognized microstate classes A–D were calculated and analyzed.

**Results:**

Compared with the activity in the corresponding functional networks of sham TENS group, the brain temporal dynamics depicted by the eyes-closed resting-state microstates of TENS group revealed significantly increased activity in functional networks involving microstate class B (MsB). Moreover, compared with that at baseline, activity in functional networks involving MsB was significantly decreased, and that involving microstate class D (MsD) was increased after sham TENS.

**Conclusion:**

Our study revealed that TENS with 1 mA or half of the maximum tolerable current may alter EEG microstate parameters in migraine patients. This study provides new insights into the treatment-related neural responses of migraine patients and a possible basis for evaluating the factors influencing migraine treatment through the use of EEG microstate analysis in the future.

## Introduction

Migraine is a very common neurobiological headache disorder (1), affecting more than one billion people (2). The prevalence of migraine is high, and its incidence has increased over the past few decades (3–5). Migraine is a leading cause of disability worldwide (6, 7) and imposes significant health and financial burdens on both the patients and their families (8).

Migraine typically alters the temporal dynamics of various brain networks. Resting-state electroencephalography (EEG) microstates are global patterns of potential topography that reflect the brief activation of several interacting components of resting-state brain networks and the states of conscious mind (9, 10). The majority of previous studies have identified four prototype microstates (labeled MsA, MsB, MsC and MsD) from EEG signals (11–13). Research suggests that the four microstates reflect auditory network (AuN), visual network (VN), salience network (SN) and dorsal attention network (DAN) respectively (14–17). In microstate time series, transition between microstates represent sequential activation of different information processing functions and neural networks (9). Consequently, resting-state EEG microstates can provide novel perspectives for investigating the pathophysiological mechanisms underlying neuropsychiatric disorders and the neural responses to treatment. Previous EEG studies have confirmed that patients with migraine have unique alterations in their resting-state EEG microstates (10, 14, 18). In migraine patients, alterations in resting-state EEG microstates are primarily reflected in the time coverage and occurrence of MsB and MsD, as well as in the transition probability between these two classes. These changes represent the abnormal function of visual cortex and attentional network at resting state (19, 20). Thus, EEG microstates may be utilized to investigate the global functional states of the brain in migraine; elucidate the neural mechanisms underlying the onset and progression of migraine from the perspective of resting-state network disruptions; and assist in the diagnosis of migraine as well as the assessment of treatment efficacy. However, no studies have yet explored the neural responses of migraine patients to therapeutic treatment through EEG microstate analysis.

A large body of evidence suggests that acupuncture has a positive clinical effect on migraine (21–23) while offering some advantages in terms of efficacy and safety over other treatment methods (24–27). A growing number of EEG studies are demonstrating that acupuncture modulates the temporal dynamic features of the EEG microstates of both healthy subjects and patients (28, 29). This implies that acupuncture could be used as a therapeutic treatment in EEG microstate studies for patients with migraine. Moreover, EEG microstate analysis has been used as a whole-brain mapping tool for investigating large-scale brain networks (28, 30); coupled with evidence of the wide-ranging effects of acupuncture on the brain and its broad spectrum of neural mechanisms of action (31, 32), we believe that acupuncture is suitable for exploring microstate alterations in the treatment of migraine. No studies, however, have described the EEG microstates related to the acupuncture treatment of migraine patients to date.

To that end, this study applied EEG microstate analysis to explore the immediate neural responses of migraine patients to acupuncture treatment. Given that acupuncture is an effective treatment for migraine and that MsB and MsD abnormalities in migraine patients exist many inconsistencies between studies (9, 10, 14, 18), we believed that acupuncture would modulate the temporal dynamic features of MsB and MsD in migraine patients and observed the direction of their change. As manual acupuncture does not allow the precise control of stimulation intensity, in this study, transcutaneous electrical nerve stimulation (TENS) over the acupoint, a traditional Chinese acupuncture therapy that is widely used in clinical practice (33), was employed as the therapeutic approach. The results of this study will enhance our understanding of the treatment-related neural responses in migraine patients.

## Methods

### Participants

This study included the data of 51 patients with migraine with or without aura from October 2022 to August 2023, who were randomly divided into two experimental groups: sham or real TENS. All patients had received diagnoses by experienced doctors according to the classification criteria of the International Headache Society (34). Preventive treatment during the interictal period is important (35), and acupuncture is frequently used for this purpose (36); therefore, all patients were in the interictal phase at the time of their participation in the study. All participants had normal or corrected-to-normal vision and self-reported being right handed. They were between 18 and 60 years old and had no history of smoking or alcohol or drug abuse. Participants were excluded if they met any of the following criteria: other types of migraine; secondary headache caused by cerebral hemorrhage, cerebral infarction, aneurysm, intracranial space occupying, an arteriovenous malformation, or other types of primary headache; mental or nervous system diseases (such as epilepsy, dizziness, cerebral infarction, cerebral hemorrhage, brain tumor, etc.); or abnormal EEG results.

All the subjects signed informed consent forms and were asked to complete the Headache Impact Test-6 (HIT-6) questionnaire before the experiment. All the participants received monetary compensation for their participation, and their data were anonymized prior to analysis. All research procedures were conducted in accordance with the Declaration of Helsinki and approved by the Ethics Committee of the Institute of Acupuncture and Moxibustion, China Academy of Chinese Medical Sciences, with the approval number S2023-10-23-1.

### Experimental design

**Figure 1:**
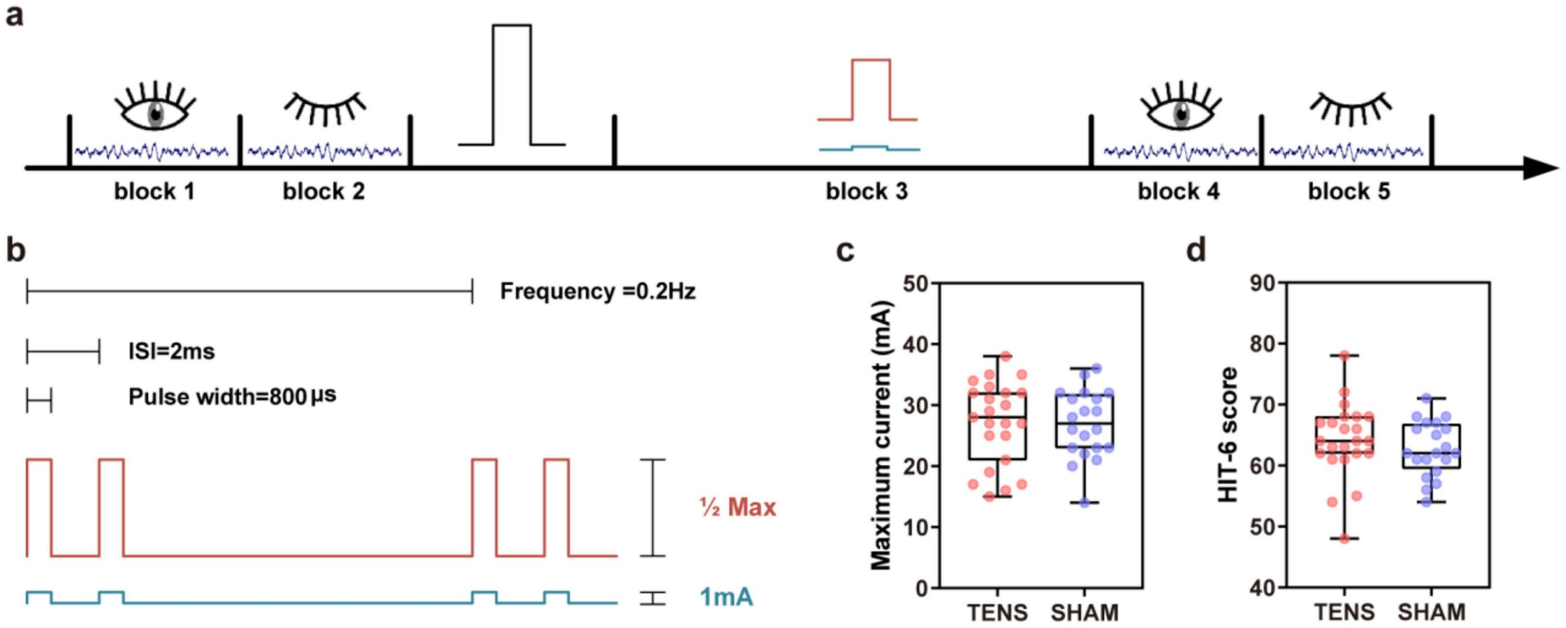
Experimental design and distributions of the maximum current intensity and HIT-6 score in the two groups. **(a)** Experimental procedure. **(b)** TENS parameters. **(c)** Maximum current intensities tolerated by the patients, shown as whisker-box plots (whisker: full range; line: median). **(d)** HIT-6 scores of the patients in the two groups, shown as whisker-box plots (whisker: full range; line: median). Comparisons of the maximum current intensities and HIT-6 scores between the real and sham TENS groups were performed with the two-tailed unpaired *t* test. ISI, inter-spike interval; Max, maximum current intensity tolerated by the patient; TENS, transcutaneous electrical nerve stimulation group; SHAM, sham group.

During the experimental sessions, the patient was seated in a comfortable chair in a quiet room. The experimental procedure consisted of 5 blocks and lasted a total of approximately 40 min (Figures 1a,b). In the first block, resting-state, eyes-open EEGs were recorded (approx. 6 min), during which the participant was instructed to fixate on a point on the wall in front of them to minimize eye movements. In the second block, the participant was asked to closed their eyes, and EEG activity was again recorded in the resting state (approx. 6 min). In the third block, the patient received TENS over the right Hegu (LI4) acupoint (approx. 10 min), which was localized according to the WHO Standard Acupuncture Point Locations in the Western Pacific Region [Hegu was one of the most commonly used acupoints for the migraine treatment and has been shown to be effective in migraine management (37, 38)]; the participants were informed that they might or might not feel the electrical stimulation depending on the current intensity used. Before receiving TENS, the maximum current intensity for each participant was determined by increasing the intensity until the maximum level tolerated was reached. For the sham TENS group, patients were stimulated with a current of 1 mA; for the TENS group, patients were stimulated with half of the maximum tolerable current. Following sham or real TENS, eyes-open and eyes-closed resting-state EEG data were again recorded in the fourth and fifth blocks, respectively (approx. 6 min each).

TENS was performed with an OSIRIS NeuroStimulator (inomed Medizintechnik GmbH, Germany). Stimulation was delivered over the right Hegu (LI4) acupoint in a bipolar fashion with an electrode connected to a high-current stimulator. The frequency was set to 0.2 Hz. Positive square wave pulses with a pulse duration of 800 μs were used. Two pulses were delivered during one burst, and ISI, the duration between two impulses in one burst, was set to 2 ms (Figure 1b).

### EEG data acquisition

**Figure 2:**
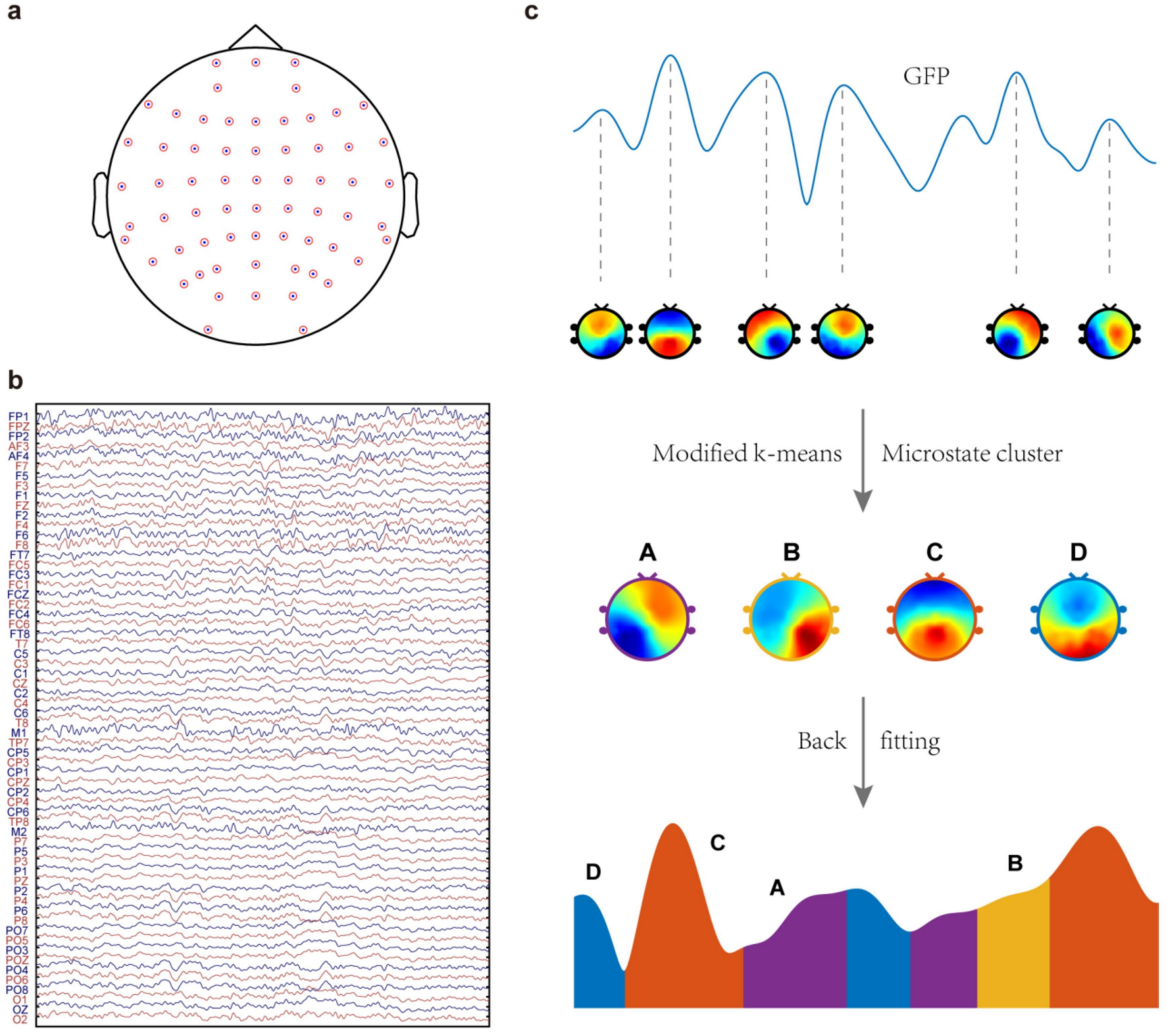
EEG acquisition and processing analysis. **(a)** Electrode locations. **(b)** An example of the EEG signal displayed by channels. **(c)** EEG microstate analysis process. GFP, global field power.

EEGs were recorded with a 64-electrode Neuroscan Quick-Cap™ connected to a SynAmps^2^ Neuroscan System (Compumedics Neuroscan™, United States) (Figure 2b). The electrodes were placed according to the International 10–20 system (Figure 2a) (39). The ground electrode was positioned between Fpz and Fz, and the reference electrode was placed between Cz and Cpz. The EEG signal was amplified with SynAmps^2^ DC amplifiers, digitized with Curry 7 software (version 7.0.8), and sampled at 20 kHz. The scalp of each participant was prepared for EEG recording via application of a conductive gel (Quick-Gel, Neuromedical Supplies^®^). The electrode impedance was generally maintained below 5 kΩ prior to data collection. The patients were asked to relax and avoid thinking any specific thoughts during the recording.

### EEG data preprocessing

The recorded data were preprocessed offline in MATLAB (version R2020b; MathWorks Inc.) using the EEGLAB toolbox (40) and custom scripts. The EEG signals were saved in the .set file format and resampled at 1000 Hz. Useless channels such as CB1 and CB2 were removed from the stored data. The resulting signals were first low-pass filtered at 70 Hz, followed by high-pass filtering at 0.5 Hz. Blink and eye movement correction was performed via independent component analysis through the CORRMAP plug-in in EEGLAB (41). Artifacts from head and body movements were rejected by visual inspection and independent component analysis.

### EEG microstate analysis

Microstate analysis was performed in MATLAB (version R2024a; MathWorks, Inc.) using the EEGLAB (40) toolbox and custom code (Figure 2c). Bad channels were interpolated first. The EEG data were then referenced to the average and bandpass filtered between 2 and 20 Hz.

The eyes-open and eyes-closed EEG signals were analyzed separately. First, the EEG data were segmented into epochs of 2 s, after which bad epochs were removed. For each patient, the same number of epochs were selected and merged to form a sequence of consecutive EEG data. Next, the EEG data from all participants were merged for microstate analysis.

For the resting-state EEG microstate analysis, the global field power (GFP) was initially extracted from the EEG signals. Considering that the local maximum (peak) of the GFP exhibits a favorable signal–to–noise ratio and stable changes in EEG topography (42), we set a minimum interval of 10 ms between consecutive GFP peaks and extracted brain topographic maps (also known as original maps) at 2000 randomly selected GFP peak locations. GFP peaks that exceeded one times the standard deviation were removed, as such peaks often contain artifacts of nonneuronal origin. All the obtained original maps were subsequently input into a modified k-means clustering algorithm (ignoring polarity) (43). We employed an optimized segmentation scheme with 1,000 iterations to obtain the best clustering. We identified 4 prototype microstates for the resting-state EEG signals, since 4 topographies has been reported to be the most common and highly reproducible for resting-state EEG signals (30, 44–46). The four optimal prototype microstates are MsA, MsB, MsC, and MsD. Next, the four prototype microstates underwent back fitting to all the EEG recordings. Each EEG time point was categorized as one of the four prototype microstates, thereby transforming the EEG signals into a microstate sequence. Finally, the microstate sequence was temporally smoothed, and segments with durations shorter than 20 ms were redistributed to the next best-fitting microstate (47). The variance in EEG activity explained by all prototype microstates (global explained variance, GEV) was reported. To quantify the microstates, the following four parameters for the microstates of each participant were computed: mean duration, occurrence per second, coverage and transition probability (9, 14, 48).

### Statistical analyses

Statistical analyses were performed in IBM SPSS Statistics (version 24) and MATLAB (version 2020b). First, subject demographics, the maximum current intensity and the HIT-6 score were compared between the groups. For the statistical comparison of categorical variables, we used the chi-square test with Fisher’s exact test. Continuous variables were first subject to the Shapiro–Wilk test to assess the normality of the distributions. Normally distributed variables were compared with the two-tailed unpaired *t* test, whereas nonnormally distributed variables were compared with the nonparametric test (Mann–Whitney *U* test).

Resting-state EEG microstate parameters with eyes open and eyes closed were analyzed separately. The normalized (% change relative to baseline) mean duration, occurrence and coverage, were compared between the real and sham TENS groups with the two-tailed unpaired *t* test or Mann–Whitney *U* test depending on the normality of the distribution of the variables. To compare the poststimulation data with the baseline data, the two-tailed paired *t* test or Wilcoxon signed-rank test was performed separately for both the TENS group and the sham TENS group, depending on the normality of the distribution of the variables. Intragroup differences between the baseline and poststimulation data for the two groups were further compared by analyzing the transition probability following the same methodology used for the mean duration, occurrence and coverage.

Given the existence of parallel microstates, we conducted false discovery rate (FDR) corrections for *p* value clusters with a MATLAB script in the statistical analyses of the microstate parameters to reduce the type I error rate and resolve the multiple comparisons problem.

## Results

We initially recruited a total of 51 patients. They were randomly and blindly assigned to TENS or sham TENS groups. Owing to the characteristics of TENS, the person administering the interventions cannot be blinded to the assignments. However, the assessors and statisticians for the data collection and analysis were blinded to the assignments. Following application of the inclusion criteria and exclusion criteria, 43 patients were included in the final analyses (23 in the TENS group and 20 in the sham TENS group). The detailed demographic characteristics of all migraine patients are shown in Table 1. There were no significant differences in sex (Fisher’s exact test, *p* = 0.092), height (Mann–Whitney *U* test, *p* = 0.591), weight (Mann–Whitney *U* test, *p* = 0.591) or age (Mann–Whitney *U* test, *p* = 0.373) between the real and sham TENS groups. In addition, we found that there were no significant differences in the maximum current intensity (two-tailed unpaired *t* test, *t*(41) = 0.143, *p* = 0.887) or the HIT-6 score (two-tailed unpaired *t* test, *t*(41) = 0.803, *p* = 0.426) between the two groups (Figures 1c,d).

**Table 1:** Demographic characteristics.

Characteristics	TENS (*n* = 23)	Sham TENS (*n* = 20)	*p* value
Sex			0.092
Male	0 (0)	3 (15)	
Female	23 (100)	17 (85)	
Height (cm)	162.00 (159.00–165.00)	160.50 (158.50–165.75)	0.591
Weight (kg)	58.00 (52.00–64.00)	54.00 (51.25–61.50)	0.591
Age (years)	32.00 (24.00–40.00)	24.50 (23.00–40.50)	0.373

Continuous variables are reported as medians (interquartile ranges). Categorical variables are reported as *n* (%).

### Cluster evaluation

**Figure 3:**
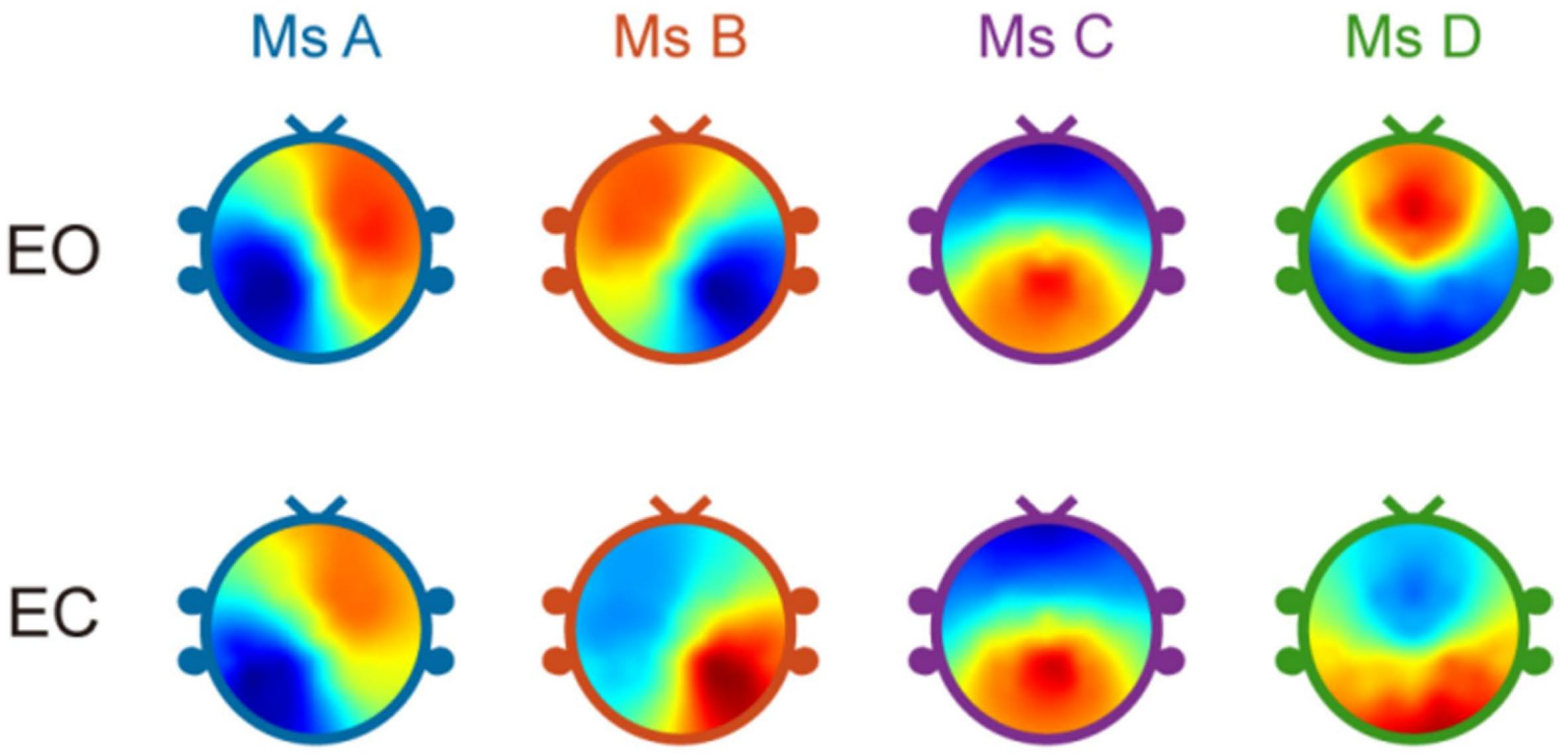
Prototype microstates with eyes open and closed. Ms., microstate; EO, eyes open; EC, eyes closed.

The eyes-open and eyes-closed resting-state EEG data were separately analyzed to explore the EEG alterations following TENS. Modified k-means cluster analysis ultimately yielded four prototype microstates from both the eyes-open and the eyes-closed EEG data (Figure 3); these microstates were strongly consistent with previous works and marked MsA, MsB, MsC and MsD (11, 12).

For the EEG data with eyes open, the average GEV of the four prototype microstates for all the subjects was 76.73% (SEM = 0.38%). The GEVs of the TENS group at baseline and poststimulation and of the sham TENS group at baseline and poststimulation were 76.73% (SEM = 0.79%), 76.70% (SEM = 1.03%), 77.02% (SEM = 0.47%) and 76.49% (SEM = 0.59%), respectively.

With respect to the EEG data with eyes closed, the average GEV of the four prototype microstates across all the subjects was 78.83% (SEM = 0.48%). The GEVs of the TENS group at baseline and poststimulation and of the sham TENS group at baseline and poststimulation were 77.76% (SEM = 1.05%), 78.32% (SEM = 1.06%), 79.48% (SEM = 0.72%) and 79.98% (SEM = 0.86%), respectively.

### Microstates with eyes open

**Figure 4:**
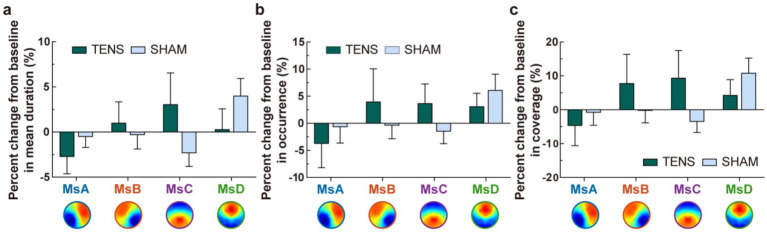
Comparison of eyes-open EEG microstate parameters between the two groups. **(a–c)** Show the percentage changes from baseline in the mean duration, occurrence, and coverage, respectively, of each prototype microstate in each of the two groups. Ms., microstate; TENS, transcutaneous electrical nerve stimulation group; SHAM, sham group.

**Figure 5:**
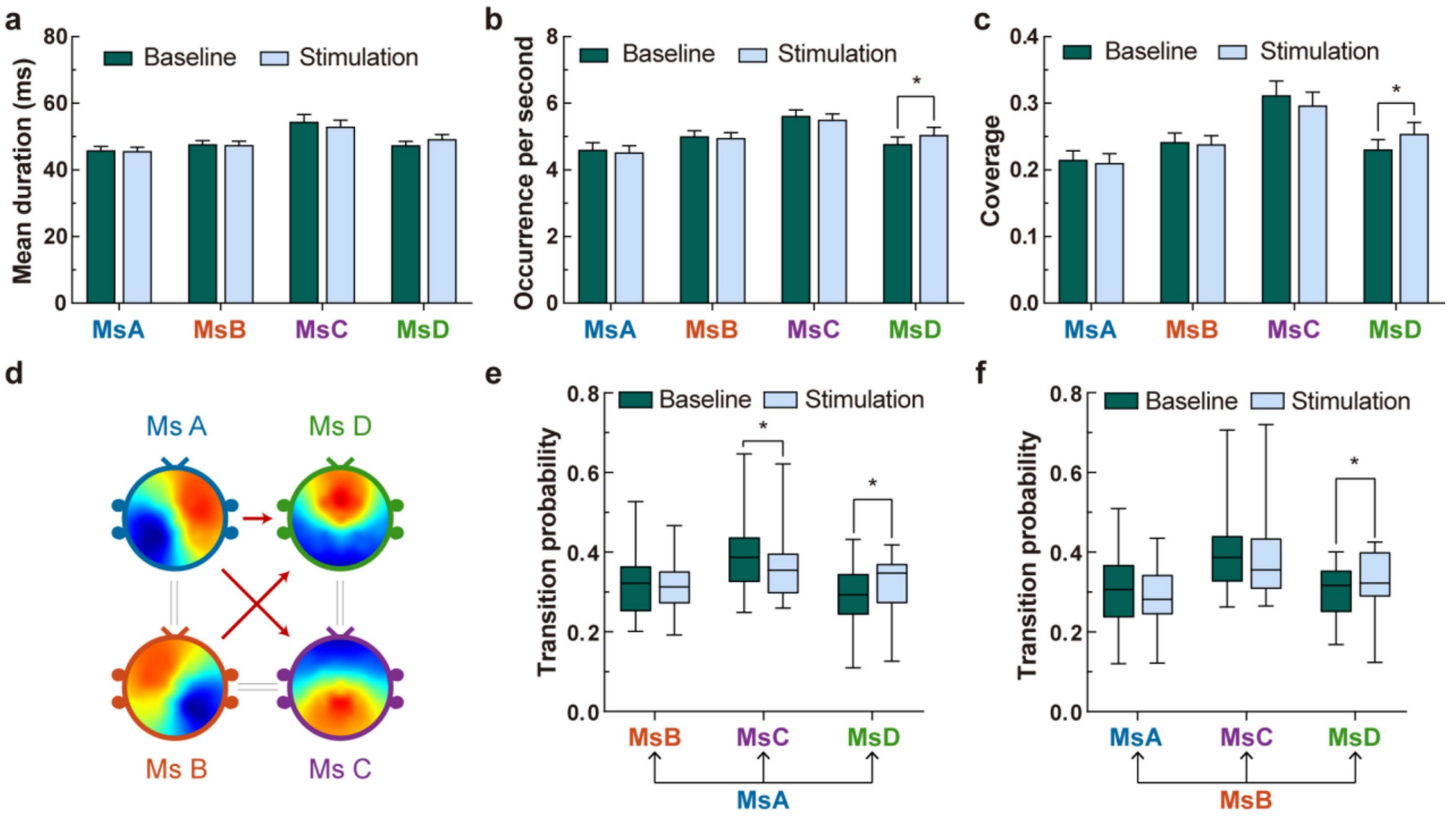
Comparison of eyes-open EEG microstate parameters between baseline and poststimulation in the sham TENS group. **(a–c)** Show the mean duration, occurrence, and coverage, respectively, of each prototype microstate at both baseline and poststimulation. **(d)** Shows the transition probabilities. Red arrows indicate that the transition from one microstate to another is significantly different between baseline and poststimulation. **(e,f)** Show the transition probabilities from MsA and MsB to others, respectively. Ms., microstate. *p < 0.05.

Regarding the percentage change from baseline in mean duration, occurrence and coverage, no differences were observed between the real and sham TENS groups (*p* > 0.05) (Figures 4a–c). Regarding the intragroup differences between baseline and poststimulation, no significant microstate changes were found following TENS (*p* > 0.05) (Supplementary Figures S1B–D). However, according to the two-tailed paired *t* test, compared with those at baseline, the occurrence (*t*(19) = −2.284, *p* = 0.034) and coverage (*t*(19) = −2.550, *p* = 0.020) of MsD were significantly greater following sham TENS (Figures 5a–c).

To further compare the poststimulation data with the baseline data, we analyzed the transition probability. Regarding the intragroup differences in transition probability between baseline and poststimulation, no significant changes were found in the TENS group (*p* > 0.05) (Supplementary Figures S1A,E–H), whereas the two-tailed paired *t* test revealed a statistically significant decrease in the transition probability from MsB and MsA to MsD and a statistically significant increase in the transition probability from MsA to MsC after sham TENS relative to baseline (*p* < 0.05) (Figures 5d–f).

### Microstates with eyes closed

**Figure 6:**
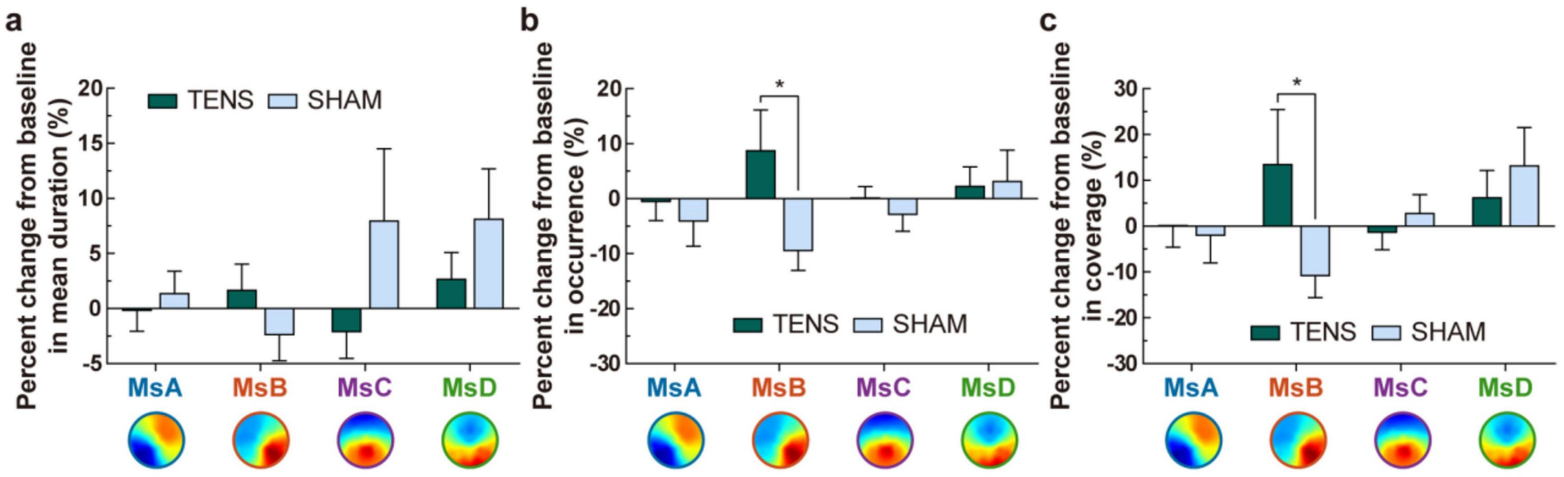
Comparison of eyes-closed EEG microstate parameters between the two groups. **(a–c)** Show the percentage changes from baseline in the mean duration, occurrence, and coverage, respectively, of each prototype microstate in each of the two groups. Ms., microstate; TENS, transcutaneous electrical nerve stimulation group; SHAM, sham group. *p < 0.05.

**Figure 7:**
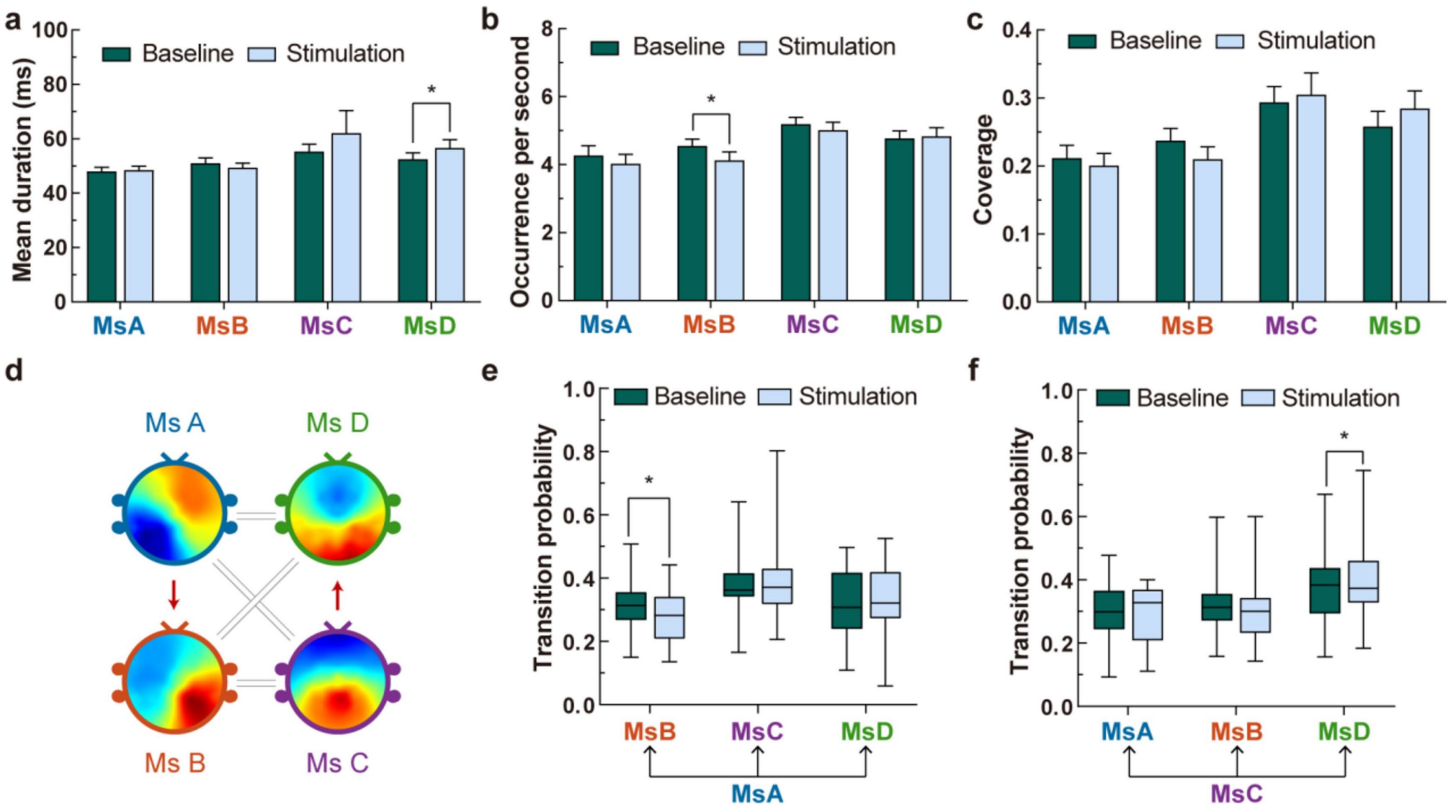
Comparison of eyes-closed EEG microstate parameters between baseline and poststimulation in the sham TENS group. **(a–c)** show the mean duration, occurrence, and coverage, respectively, of each prototype microstate at both baseline and poststimulation. **(d)** shows the transition probabilities. Red arrows indicate that the transition from one microstate to another is significantly different between baseline and poststimulation. **(e,f)** show the transition probabilities from MsA and MsC to others, respectively. Ms., microstate. *p < 0.05.

With respect to the percentage changes from baseline in the three microstate parameters, Mann–Whitney *U* tests revealed a significant increase in the occurrence (*p* = 0.017) and coverage (*p* = 0.049) of MsB in the TENS group relative to the sham TENS group, whereas there were no differences in the mean duration between the groups (*p* > 0.05) (Figures 6a–c). Regarding the intragroup differences between baseline and poststimulation, no significant microstate changes were observed following TENS (*p* > 0.05) (Supplementary Figures S2B–D). However, the Wilcoxon signed-rank test revealed a statistically significant increase in the mean duration of MsD (*p* = 0.037) and a decrease in the occurrence of MsB (*p* = 0.026) following sham TENS relative to baseline (Figures 7a–c).

To further compare the poststimulation data with the baseline data, the microstate transition probabilities were analyzed. Regarding the intragroup differences in transition probability between baseline and poststimulation, no significant changes were observed for the TENS group (*p* > 0.05) (Supplementary Figures S2A,E–H), whereas compared to those at baseline, the probability of transition from MsA to MsB decreased (two-tailed paired *t* test, *t*(19) = 2.160, *p* = 0.44), and the transition from MsC to MsD increased after sham TENS (Wilcoxon signed-rank test, *p* = 0.015) (Figures 7d–f).

## Discussion

A previous clinical trial provided strong evidence that acupuncture is effective in managing migraine (49). However, few studies have focused on the neural response of migraine patients to acupuncture through EEG. To our knowledge, this is the first study to investigate the neural effects of acupuncture treatment on EEG microstates in migraine patients. In this study, through EEG microstate analysis, we found that TENS treatment changed the temporal dynamic features of brain networks in migraine patients with respect to those of the sham TENS group. In addition, the microstate parameters of migraine patients were altered after sham TENS. Previous research has shown that migraine patients exhibit abnormalities in their EEG microstates, which may reflect the disorders in their brain networks (18, 50). We therefore propose that alterations in EEG microstates could aid in interpreting the treatment-related neural responses of migraine patients.

Previous studies have suggested that EEG microstates could be regarded as the “atoms” of brain activity, as they may represent the basic building blocks of brain information processing (30, 51, 52). Research has confirmed that the coverage and occurrence of MsB and MsD were increased in migraine patients, as well as the transition probability between these two microstates (9, 10, 18); however, Zhou et al. found reduced coverage, occurrence of MsD and transition probability from MsB to MsD (14). In any case, patients with migraine demonstrate alterations in the parameters of MsB and MsD. Similarly, the alterations seen in our results tended to be related to these prototype microstates. Analysis of the resting-state, eyes-closed EEG data revealed that the percentage change from baseline in the occurrence and coverage of MsB in the TENS group (TENS with half of the maximum tolerable current) was greater than that in the sham TENS group (1 mA TENS). When this analysis was repeated for the resting-state, eyes-open EEG data, the occurrence and coverage of MsD as well as the probability of transition from MsB to MsD were found to be greater after 1 mA TENS than at baseline. Moreover, changes in other microstate parameters, such as the mean duration of MsD and the transition probability from MsA to MsC, were also observed in this study. Taken together, these findings suggest that the EEG microstates, especially MsB and MsD, could provide neuroimaging evidence for elucidating the treatment-related neural responses of migraine patients. Furthermore, these results provide a potential basis for selecting the optimal stimulation intensity for TENS treatment of migraine; suggest that EEG microstate parameters may be indicators of therapeutic efficacy in migraine patients; and suggest that microstates may be further utilized to design targeted treatments in clinical setting.

In the present study, the EEG microstates were analyzed separately for the eyes-open and eyes-closed states. First, EEG data obtained with the eyes open and eyes closed may be different and may not be comparable and therefore should not be combined for analysis. Research has shown that the microstate parameters substantially differ between eyes-open and eyes-closed conditions (53); for example, the visualization condition has been shown to affect the coverage of MsA (54). Second, some migraine patients may experience visual discomfort and visual processing disorders (14, 55), and the microstate may influence the perception of visual stimuli; a study revealed that the EEG microstates prior to stimulus onset affect perceptual awareness of visual stimuli near the threshold of awareness (56).

In a comparison study between real and sham TENS, we found no intergroup differences in the percentage change from baseline in three microstate parameters (mean duration, occurrence per second and coverage) for resting-state EEG with eyes open, whereas TENS resulted in an increase in the percentage change from baseline in the occurrence and coverage of MsB for resting-state EEG with eyes closed. Many studies have reported an association between MsB and the visual resting-state network (15, 57). Visual input can influence MsB, and the occurrence and coverage parameters of this microstate are increased in the eyes-open condition (53). In the present study, eyes open condition might have led to the alterations in MsB. However, no differences in the parameters of the microstates derived from resting-state, eyes-open EEG were detected between the real and sham TENS groups, possibly because the visual inputs to the two groups were the same. In the eyes-closed condition, patients received stimulation without visual input, so the influence of visual processing on MsB was no longer dominant. However, there were some differences in MsB derived from resting-state, eyes-closed EEG, suggesting that acupuncture may modulate VN function in migraine patients. Previous studies have reported anatomical alterations of the visual motion processing network and abnormal functional connectivity of the visual primary sensory cortex network in migraine (58, 59). These changes and abnormalities are reflected in the occurrence and coverage of MsB. The results in the eyes-closed resting state in this study further confirmed that acupuncture may exert its therapeutic effect by modulating abnormalities in VN in migraine patients. This speculation is supported by previous research in acupuncture which has converged to reveal that acupuncture modulates the neural activity of VN (60). Consequently, acupuncture may modulate VN in migraine patients, which manifests in MsB.

In the comparison of the EEG microstates before and after TENS treatment, we found no differences in either the eyes-open or eyes-closed condition in the TENS group. Research suggests that neural activity in the brain is reduced when stimulus input aligns with sensory predictions (61). In this study, the maximum tolerated current intensity for each patient were assessed prior to the intervention; it is thus reasonable to infer that the bodies of patients had become accustomed to, and could predict, stimuli at half of the maximum tolerable current, which may have resulted in the lack of changes in the microstates of the TENS group. Alternatively, this result could be due to an insufficient current intensity, but further experiments are needed to explore this possibility. In the comparison of the EEG microstates before and after sham TENS treatment, we found an increased occurrence and coverage of MsD in the eyes-open condition and an increased mean duration of MsD and a decreased occurrence of MsB in the eyes-closed condition. Similar to the results of the intergroup comparisons, the alterations in MsB derived from resting-state, eyes-closed EEG may also be related to the regulation of VN by sham acupuncture, but further investigations are required to validate this hypothesis. In addition, previous studies have shown that MsD is related to activities in the DAN which reflects reflexive aspects of attention (15, 62). Migraine patients have attention impairment, and a reduction of MsD leads to the attentional deficits (14, 63). The occurrence, mean duration and coverage of a particular prototype microstate are, respectively, interpreted to reflect the tendency of its underlying neural generators to be activated, the stability of its underlying neural assemblies, and the relative time coverage of its underlying neural generators (9). It is therefore reasonable to infer that the increase in these parameters of MsD following 1 mA TENS may indicate the increase in the likelihood of neural activation of DAN and the intensity of its neural activity, thereby enhancing attention in migraine patients. However, this hypothesis requires further research. Consequently, the increase in MsD parameters under these two conditions may be associated with alterations in attention and sham acupuncture may regulate DAN in migraine patients.

There are several limitations in this study. First, this study examined only the immediate microstate responses to acupuncture in migraine patients, with the stimulation performed just once; therefore, its influence on the EEG microstates may also be limited. Furthermore, we did not distinguish between migraine patients with and without aura, preventing further subgroup analysis to determine whether the reported findings are driven by a specific clinical phenotype. Moreover, patients were only stimulated with 1 mA or half of the maximum tolerable current. We did not consider other stimulation intensities and therefore could not analyse their effects on alterations in EEG microstates.

## Conclusion

In conclusion, compared with 1 mA TENS, TENS with half of the maximum tolerable current increased the eyes-closed resting-state microstate parameters of migraine patients. In addition, the resting-state microstate parameters of migraine patients changed after 1 mA TENS. The increase in the resting-state microstates was observed mainly in the MsD; the decrease in them was noted primarily in the MsB. This study provides new insights into the treatment-related neural responses of migraine patients and provides a possible basis for evaluating the factors influencing migraine treatment through the use of EEG microstate analysis in the future.

## Data Availability

The raw data supporting the conclusions of this article will be made available by the authors, without undue reservation.
