# Supplementary material for: EEG microstate responses of migraine patients to transcutaneous electrical nerve stimulation
Source: Front Neurol. 2026 Jul 29;17:1883163. doi: 10.3389/fneur.2026.1883163 (PMC13461500; doi:10.3389/fneur.2026.1883163)
Supplement: Supplementary file 1 [file Table_1.DOCX]

Supplementary Materials for

**EEG microstate responses of migraine patients to transcutaneous electrical nerve stimulation (TENS)**

**The PDF file includes:**

Figs. S1 to S2

**Figure S1. Comparison of eyes-open EEG microstate parameters between baseline and poststimulation in the TENS group.** **A** shows the transition probabilities. **B**, **C** and **D** show the mean duration, occurrence and coverage, respectively, of each prototype microstate at both baseline and poststimulation. **E**‒**H** show the transition probabilities from MsA, MsB, MsC and MsD to others, respectively. Ms, microstate

**Figure S2. Comparison of eyes-closed EEG microstate parameters between baseline and poststimulation in the TENS group.** **A** shows the transition probabilities. **B**, **C** and **D** show the mean duration, occurrence and coverage, respectively, of each prototype microstate at both baseline and poststimulation. **E**‒**H** show the transition probabilities from MsA, MsB, MsC and MsD to others, respectively. Ms, microstate
